# Dietary Patterns of Pregnant Women and Their Association with Diet Quality Measures: A Comparative Analysis

**DOI:** 10.3390/nu16111736

**Published:** 2024-06-01

**Authors:** Andreea-Maria Mitran, Alina Delia Popa, Andreea Gherasim, Otilia Nita, Laura Mihalache, Lidia Iuliana Arhire, Dumitru Gafitanu, Monica Hancianu, Oana Cioanca

**Affiliations:** 1 Faculty of Medicine, “Grigore T. Popa” University of Medicine and Pharmacy, 16 Universitatii Street, 700115 Iași, Romania; mitran.andreea-maria@email.umfiasi.ro; 2 Department of Nursing, Faculty of Medicine, “Grigore T. Popa” University of Medicine and Pharmacy, 16 Universitatii Street, 700115 Iași, Romania; 3 Department of Internal Medicine II, Faculty of Medicine, “Grigore T. Popa” University of Medicine and Pharmacy, 16 Universitatii Street, 700115 Iași, Romania; andreea.gherasim@umfiasi.ro (A.G.); otilia.nita@umfiasi.ro (O.N.); laura.mihalache@umfiasi.ro (L.M.); lidia.graur@umfiasi.ro (L.I.A.); 4 Department Maternal and Child Health, “Grigore T. Popa” University of Medicine and Pharmacy, 16 Universitatii Street, 700115 Iași, Romania; dumitru.gafitanu@umfiasi.ro; 5 Faculty of Pharmacy, “Grigore T. Popa” University of Medicine and Pharmacy, 16 Universitatii Street, 700115 Iași, Romania; monica.hancianu@umfiasi.ro (M.H.); oana.cioanca@umfiasi.ro (O.C.)

**Keywords:** dietary patterns, diet quality, pregnancy, maternal nutrition, nutritional epidemiology

## Abstract

Healthy dietary patterns during pregnancy are crucial for ensuring maternal and foetal health outcomes. Numerous methodologies exist for assessing the diet of pregnant women, including dietary patterns and various appraisal tools of diet quality. This study aimed to assess the dietary patterns and diet quality of pregnant women and to investigate the relationship between dietary patterns, diet quality estimators, and the adequacy of nutrient intake. EPIC FFQ was applied to a sample of 251 pregnant women, and questionnaires were interpreted with the FETA program. Dietary patterns were then determined by means of principal component analysis. Our results showed a substantial association between dietary patterns and total diet quality, as measured by the Diet Quality Index for Pregnancy (DQI-Pc), PURE Healthy Diet Score, and FIGO Diet Quality Score. We also found correlations between certain dietary patterns and particular nutrient intakes recommended by the European Food Safety Authority during pregnancy. The most deficient intake was registered for iron (86.1%), zinc (87.3%) and magnesium (79.3%), posing a threat to normal bone development, anaemia prophylaxis, and immune status. These results highlight the importance of assessing and understanding eating habits during pregnancy in order to achieve optimal outcomes for both the mother and the foetus.

## 1. Introduction

Proper nutrition during pregnancy holds great significance, as it not only affects the well-being of the expectant mother and the developing foetus but also has implications for future generations. The dietary choices made at this time have a ripple effect that extends well into the future of the offspring [1], emphasising the need to understand and prioritise healthy eating behaviours during pregnancy.

Various studies have demonstrated that diet can influence the progression of pregnancy through intricate mechanisms [2]. Adequate intake of nutrients is dependent on a balanced and nutritious diet. Meeting recommended targets of essential nutrients such as folic acid, iron, calcium and omega-3 fatty acids is particularly important during pregnancy. Normal neural tube development is dependent on sufficient folic acid levels. Folic acid deficiency during embryogenesis has been shown to cause neural tube defects [2]. Adequate iron intake is essential in supporting the increased production of red blood cells in pregnancy. Thus, the complications associated with iron deficiency, such as increased maternal illness, low birth weight, prematurity and intrauterine growth restriction could be prevented [3]. Demands for calcium and vitamin D also increase to ensure the foetus’ proper bone development while maintaining maternal bone health [4]. Inadequate calcium intake during pregnancy can lead to restricted intrauterine growth, poor bone mineralisation, preterm birth and preeclampsia [5].

Diet quality scores (Healthy Eating Index (HEI), Prospective Urban Rural Epidemiology Healthy Diet Score (PURE), and Diet Quality Index (DQI) reflect the level of adequacy of certain diets in terms of compliance with general recommendations for specific populations. These scores offer a global evaluation in terms of variety and moderation of populations’ food intake, adding important information to those resulting from the reported intake of certain nutrients or food groups. They aim to realise an a priori broader description of dietary intake. Dietary pattern analysis is an a posteriori evaluation of the main trends in food intake and may complete the image realised by the diet characterisation using the quality scores [6]. Moreover, dietary patterns may better estimate health-related outcomes than consumption of foods or nutrients in isolation. Dietary patterns define associated food groups by intercorrelated dietary variables and are derived based on an empirical approach using statistical methods including principal component analysis (PCA) or cluster analysis [7].

Diet quality during pregnancy was associated with lower risks of gestational diabetes mellites, gestational hypertension, preeclampsia, and preterm delivery [8]. Furthermore, healthy eating during pregnancy, evaluated with HEI, was related to more favourable glucose–insulin homeostasis and lipid profiles in male offspring [9] as well as adequate body composition and reduced obesity risk during childhood [10]. Maternal dietary patterns were also related to maternal outcomes, childhood growth and obesity risk [11]. 

Given the importance of maternal diet over the course of pregnancy, diet quality, dietary patterns and nutrient intake of pregnant women have become a prominent area of research. The use of both approaches, dietary indices (a priori) and dietary patterns (a posteriori), to evaluate populations’ diets may provide a more insightful evaluation. Only a few studies have targeted the relationship between dietary patterns and overall diet quality in pregnant women [12].

The literature concerning the eating habits of Romanian pregnant women is scarce. Thereupon, this study aimed to (i) assess dietary patterns and diet quality using the Diet Quality Index for Pregnancy among pregnant women; (ii) investigate the relationship between dietary patterns, dietary quality, and the adequacy of nutrient intake as recommended by the European Food Safety Authority for dietary reference values (DRV) for pregnancy. 

## 2. Materials and Methods

### 2.1. Study Design

We conducted an observational study on a convenience sample of 251 pregnant women hospitalised before delivery, between January and July 2023, at the “Elena Doamna” Obstetrics and Gynaecology Hospital in Iasi. Women who refused to participate, under 18 years old, with twin pregnancies, obstetrical pathologies, psychiatric disorders, or other diseases that could affect the ability to provide accurate responses were excluded from our sample. Fifteen pregnant women declined to participate in the study.

The sample size was estimated with G*Power 3.1, assuming a medium effect size (f_2_ = 0.15), a level of significance (α) of 0.05, and a statistical power (1 − β) of 0.80 [13]. The sample size required for logistic regression with 10 predictors was estimated to be 187 participants. A sample size of 200 participants was estimated to be required to compare significant differences between 2 groups [14].

### 2.2. Data Collection

Socio-demographical data (age, area of residence, marital status, education, parity, income), smoking status, and data on the provision of nutritional counselling during pregnancy by healthcare professionals were collected through direct interviewing. The intake of folic acid, iron and multivitamins, and mineral supplements during pregnancy were reported in terms of name and duration of administration expressed in months. Food frequency and portion sizes were assessed using the EPIC-Norfolk Study Food Frequency Questionnaire (FFQ), which is available online [15]. The questionnaire was previously translated and validated on the Romanian population [16]. The Epic-Norfolk Study FFQ estimates the food intake over the previous year. This semiquantitative FFQ estimates the intake of 130 types of foods and drinks. Participants indicate the frequency of consumption of these items, choosing from nine options ranging from “never or less than once/month” to “6+ times per day”. An average serving size is assigned for each food and is appreciated through common portions or commonly used household measures. An additional set of questions registers the type of breakfast cereals, milk, meat, and fat used for cooking to realise more accurate estimations of fat intake. Data from participants with implausible energy intake (<800 kcal/day or >3500 kcal/day) were excluded from the analysis.

### 2.3. Adequate Nutrient Intake and Diet Quality DQI-P

Nutrient intake adequacy was established according to the recommendations of European Food Safety Adequacy for dietary reference values (DRV) for pregnancy [17]. Adequate intake was considered as ingestion of a certain nutrient exceeding two-thirds of its DRV to mitigate the overestimation bias associated with the FFQ [18]. The nutrient intake provided by different supplements was not included in the analysis of the relationship between dietary patterns and diet quality. Nutrient intake was adjusted to the energy intake according to the Willet residuals method [19]. 

The diet quality index for pregnancy (DQI-Pc) [20] is a continuous measure that contains six components: recommended servings of grains, fruits, and vegetables; recommended intake of folate, iron, and calcium; and recommended energy intake from fat. Each DQI-Pc component score can range from 0 to 10.0, except for the fruits and vegetables, which is scored out of 20.0. The sum of DQI-Pc components produces a total score out of 70.0, which is transformed into a percentage.

The Prospective Urban Rural Epidemiology Healthy Diet Score (PURE) is a tool that reflects the quality of diet assessed in epidemiological studies and takes into consideration the number of servings of vegetables, fruits, legumes, nuts, fish, and dairy products. Each component receives a score of 0 or 1, according to the achievement of the recommendations for each food group. The sum of the scores represents the PURE Healthy Diet Score, which can vary between 0 and 6 [21]. 

International Federation of Gynaecology and Obstetrics (FIGO) diet quality score (FIGO-DQS) was suggested by Tsoi et al. [22] and is similar to a short FFQ composed of 6 food groups: meat and poultry, fruit and vegetables, fish, dairy products, whole grain carbohydrates, and packaged foods. The adequate intake for each group is scored with 1 point and inadequate intake with 0 points. The FIGO-DQS score can range between 0 and 6, with higher scores representing a better diet quality.

### 2.4. Dietary Pattern Construction

**Figure 1:**
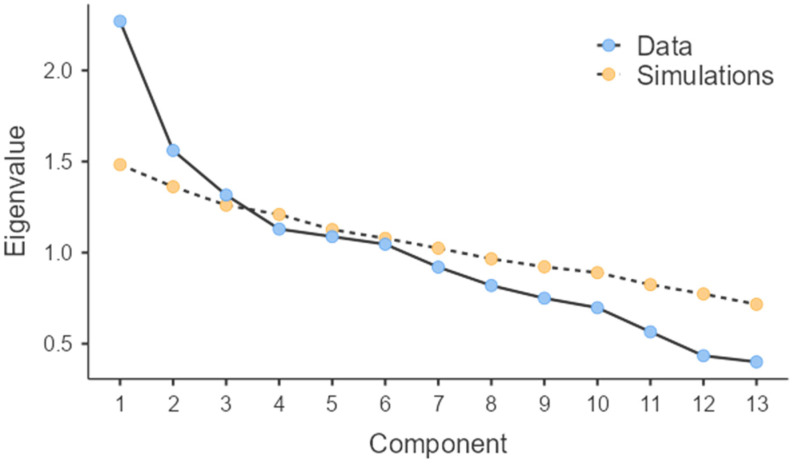
Scree plot of dietary patterns.

Using principal component analysis, we assessed dietary patterns within the 13 food groups. The suitability of factor analysis was determined with the Keyser–Meyer–Olkin (KMO) test and the Bartlett test. A KMO value exceeding 0.5 is considered acceptable. We only considered components meeting an eigenvalue above 1.3. Factor loadings above 0.25 were considered significant. In our analysis, the KMO value was 0.562. We identified three components accounting for 39.6% of the overall variance, i.e., vegetarian, modern, and prudent. The vegetarian pattern was marked by a substantial intake of cereals, cereal products, fats and oils, nuts, and seeds. At the same time, the modern pattern was defined by a significant intake of potatoes, fats, oils, soft drinks, meat, meat products, sugar, preserves, and snacks. Finally, the prudent pattern was marked by an important consumption of vegetables, fruits, soups, sauces, milk, and dairy products (Table 1, Figure 1).

### 2.5. Statistical Analysis

Statistical analysis was performed with R Studio (version 4.3.2). Descriptive analysis was used to compute the mean, median, minimum, and maximum values, standard deviation, standard error, 95% confidence interval, and frequencies. 

We used binary logistic regression to assess the association between dietary patterns (independent variable) and nutrient adequacy (dependent variable). We test this association using multiple logistic regression in a model that included maternal age, area of residence, income, formal education, parity, and nutritional education during pregnancy to control for these potential confounding factors.

### 2.6. Ethics

The current study was conducted following the Declaration of Helsinki and approved by both “Elena Doamna” Hospital of Obstetrics and Gynaecology, Iasi (1373/14 February 2023) and the Ethics Committee of Grigore T. Popa University of Medicine (348/28 September 2023) and Pharmacy. Informed consent was obtained from all subjects involved in the study.

## 3. Results

Participants’ average age was 27.58 years old, ranging from 18 to 52 years old, with 75% of women being younger than 31 years old. Regarding formal education, 33.9% (*n* = 85) of women had elementary or secondary education, 31.5% (*n* = 79) had completed high school, and 27.1% (*n* = 68) obtained a university degree. The number of multiparous women was low (18.7%), with a median number of pregnancies of 1, ranging between 1 and 13. Most women (66.5%) declared taking multivitamins and mineral supplements during pregnancy, although 85.3% of them did not receive nutritional advice from a healthcare professional. Folic acid supplementation was reported by only 8% (*n* = 20), while other nutritional supplements were used by 33.5% (*n* = 84) of women during pregnancy. Most women (61%) had a normal weight before pregnancy and 58.19% had adequate gestational weight gain (GWG). A small proportion of participants (15.9%) had inadequate weight gain during pregnancy, while an excessive GWG was registered in 23.2% (*n* = 57) of cases (Table 2).

The diet quality index for pregnancy (DQI-Pc) ranged between 26.1% and 93.1%, with a mean value of 53.3% (median 52%). Women in the upper quartile of DQI-Pc had a score higher than 59.7% (Table 3).

There was a significant correlation between the diet quality scores, which may be explained by the similarities of their components, especially between PURE and FIGO-DQS scores (Table 4). A strong positive relationship was noticed between the PURE score and DQI-Pc (Spearman’s rho 0.679; *p* < 0.001) and, respectively, between PURE and FIGO-DQS (Spearman’s rho 0.538; *p* < 0.001). DQI-Pc had a weaker but significant correlation with FIGO-QS (Spearman’s rho 0.393; *p* < 0.001).

A relatively small proportion of the women included in our sample had an adequate intake of vegetable and fruit servings. Furthermore, the intake of important nutrients included in the DQI-P was lower than the recommendations for pregnancy. Most women did not meet an intake higher than 50% of RDA for folic acid (91.2%) or iron (79.2%) (Table 4). The PURE Healthy Diet Score was relatively low in our sample, with 75% of women scoring below three points. The most deficient intake was registered for fruits, vegetables, nuts, and fish. A recommended number of servings was observed more frequently for legumes and dairy. A similar situation was registered when FIGO-DQS evaluated the quality of the diet, with 75% of women scoring below four points. The food groups with more frequent inadequate intake were fish, whole grains and packaged foods (Table 4).

Regarding micronutrients, the most deficient intake was registered for zinc (87.3%), magnesium (79.3%), and iron (86.1%). The proportion of pregnant women with an intake within the limits of DRV was higher for selenium (78.9%), iodine (69.3%), and calcium (73.7%). Only 6.4% of women had a diet that provided an adequate intake of folate during pregnancy. A low proportion of women had an adequate intake of vitamin B1 (37.8%), B6 (42.2%), C (31.9%), E (47%), and A-retinol equivalents (29.9%). A higher proportion of women had a dietary intake as recommended of vitamin D (66.5%) and vitamin B12 (81.7%) (Table 5).

Women with a prudent dietary pattern were more likely to have an adequate intake of magnesium, sodium, folate, niacin, vitamin A, vitamin A-retinol equivalents, vitamin B1, B12, B6, C, and PUFAs compared to those with a vegetarian pattern. This relationship persisted after adjustment for confounders (age, area of residence, income, formal education, parity, and dietary advice) for magnesium, sodium, niacin, vitamin A, vitamin A-retinol equivalents, vitamin B1, B6, and vitamin C. The vegetarian pattern was more likely to be associated with an adequate starch and cholesterol intake compared to the prudent pattern. Furthermore, the modern pattern was associated with a higher likelihood of an adequate intake of magnesium, niacin, vitamin A, B1, B6, and vitamin C as compared to the vegetarian pattern. Women with prudent patterns were more likely to have an adequate intake of vitamin A retinol as compared to the modern dietary pattern (Table 6).

A significant relationship was noticed between DQI-Pc and dietary patterns. Women with a prudent pattern were more likely to have a DQI-Pc score in the 75th quartile than those with a modern pattern (OR = 6.027) or those with a vegetarian pattern (OR = 4.812). There were no differences between modern and vegetarian patterns regarding the relationship with the quality of the diet (Table 6). Participants with a prudent pattern were more likely to have a better PURE and FIGO-DQS score (above the 75th percentile) as compared to those with modern or vegetarian dietary patterns. These relationships were maintained after adjusting for socio-demographic factors, parity and the presence of nutritional advice during pregnancy (Table 6). 

## 4. Discussion

Addressing the whole diet, rather than individual foods or nutrient intakes, has established itself as a powerful epidemiological instrument when studying the role of nutrition [23]. Exploring dietary patterns has been shown by multiple reviews to be a valuable predictive tool in pregnancy [24,25,26]. According to a recently published meta-analysis, the majority of observational studies on pregnant women identified two categories of dietary patterns: an unhealthy pattern, which is characterised by an increased intake of processed meat, fatty foods, and refined carbohydrates, and a healthy pattern, which is characterised by a diet high in whole grains, fruits, vegetables, and lean meat. The healthy patterns were significantly associated with a reduced risk of preterm birth, while unhealthy patterns were associated with a lower birth weight [24]. Dietary patterns have also been linked to maternal adiponectin and leptin concentrations, with Western patterns being inversely associated with adiponectin and positively associated with leptin concentrations [27].

Maternal diets high in processed foods, sugar, sweets, soft drinks and refined grains have been linked with lower birth weights or a higher risk of small-for-gestational-age babies. A recent meta-analysis found that higher consumption of milk and milk products was associated with better infant outcomes, such as a lower risk of small-for-gestational-age babies and greater birth weight and length [26]. A pattern high in cereals, coffee and tea, and processed fats was associated with inadequate (both low and excessive) birth weight, while high consumption of sweets and snacks was linked with lower chances of inadequate maternal weight gain [28]. Maternal intake of dietary groups may also have a differential effect on in-utero development. High intake of junk foods has been associated with a significantly increased birthweight at the 95th quantile, while increased consumption of vegetables was associated with a reduction in birthweight [29].

Using PCA we identified three main dietary patterns: prudent, modern and vegetarian. The prudent pattern consisted of high intakes of vegetables, fruit, soups and sauces, and milk and milk products. The modern dietary pattern was characterised by high consumption of potatoes, fats and oils, non-alcoholic beverages, meat and meat products and sugars, preserves and snacks. Finally, the vegetarian pattern implied high intakes of milk and milk products, fats and oils, cereals and cereal products, nuts and seeds. 

Principal Component Analysis (PCA) stands out as a potent methodological tool due to its capacity to effectively examine extensive datasets obtained from food frequency questionnaires (FFQs). By transforming nutrient datasets into discernible patterns, PCA facilitates enhanced visualisation and interpretation of dietary behaviours across the entire sampled population. Several studies have confirmed PCA as one of the most efficient methods of nutritional investigation. Its main advantages include simplifying complex data efficiently, reducing variables while retaining information [30], and improving interpretability [31].

Diet quality refers to a dietary pattern or to the degree of variety across the food groups that define a dietary pattern, relative to the recommendations included in dietary guidelines [32]. Diet Quality Indices (DQIs) are assessment tools used to quantify the quality of dietary intake by scoring food and/or nutrient intakes as they relate to dietary guidelines [32]. Given the complex nutritional needs of pregnant women, targeted DQIs were created, such as the Canadian Diet Quality Index for Pregnancy (DQI-Pc) [20]. 

In our sample, DQI-Pc ranged from 26.1% to 93.1% (median 52%). The prudent pattern had a higher association with DQI-Pc. Furthermore, most of the participants had an unhealthy diet according to the PURE Healthy Diet Score. Participants with a prudent pattern were more likely to have a better PURE score (above the 75th percentile) compared to those with modern or vegetarian dietary patterns. These relationships were maintained after adjusting for socio-demographic factors, parity and the presence of nutritional advice during pregnancy.

Similar DQIs used to assess pregnant women’s diets have found an association between diet quality and pregnancy outcomes. A higher DQI has been associated with a higher newborn size and lower risks of low birth weight and small-for-gestational-age babies [33,34]. Poor maternal diet quality was also associated with an increased risk of large-for-gestational-age babies, independent of maternal obesity [35]. Moreover, diet quality during pregnancy was associated with the diet quality of the offspring at 14 years old [36], further emphasising the importance of maternal diet across generations. Greater adherence to dietary guidelines during pregnancy may also improve male offspring’s metabolic profile, as an inverse association between maternal diet quality and offspring insulin, glucose, adiponectin and HOMA-IR was observed [9].

In comparison to the vegetarian group, the prudent pattern was more likely to ensure an adequate intake of magnesium, sodium, vitamins A and C, as well as group B vitamins, cholesterol, and polyunsaturated fatty acids (PUFAs). Compared to the vegetarian group, the modern dietary pattern ensured a higher likelihood of meeting the recommended intake for magnesium, B vitamins, vitamin A, and cholesterol. A prudent pattern was more likely to meet vitamin A requirements than the modern pattern.

In the present sample, we identified a concerning trend—only 6.4% of women met the necessary dietary requirements for folate, and only 8% reported taking folic acid supplements. In addition, 66.5% of women reported using pregnancy multivitamins and multimineral formulations, in the form of food supplements. These results align with those of a 2021 Romanian study, which found that 31% of pregnant women did not use any type of supplement during pregnancy [37]. This is a worrisome finding, as the detrimental effects of folate deficiency during pregnancy have been observed since 1991 [38]. It is worth noting that Romania does not practice flour fortification, making the need to increase folate intake even more important. The risks of inadequate folate intake extend beyond preventing neural tube defects. 

The increased rate of erythropoiesis demands an increased intake of iron, folate, and cobalamin. Although the observed intake of vitamin B12 was satisfactory (81.7%), iron intake was significantly low, with only 13.9% of women meeting the requirements. The developing brain of the foetus is at particular risk of iron deficiency, with research linking low maternal iron intake to autism, schizophrenia and abnormal brain structure in the offspring [3]. Correlated with the unsatisfactory folate intakes, such low iron intakes raise concerns regarding successful prophylaxis of anaemia in pregnancy. Besides its important role in the prevention of neural tube defects and anaemia, folic acid may also play a role in preventing foetal structural anomalies such as congenital heart defects and oral clefts, and possibly preterm births [39].

Only 12.7% of women had satisfactory zinc intake, which is among the lowest observed intakes. This aligns with Benedictis et al.’s estimate that 87% of women globally have improper zinc intake, including 47% of pregnant women [40]. Zinc plays a role in many physiological processes, including cellular division, nucleic acid metabolism, protein synthesis and immune system function [41], making it indispensable during pregnancy. Studies have shown that zinc deficiency during pregnancy increases the risk of low birth weight and small-for-gestational-age babies [42]. Animal research has shown that insufficient zinc levels during pregnancy have long-term effects on the offspring, leading to impaired immune function that persisted for three generations [43]. Human mothers with acrodermatitis enteropathica, an inherited disease that causes profound zinc deficiencies, commonly report poor pregnancy outcomes, such as congenital malformations and foetal losses [44]. It is important to note that smoking mothers are at an even greater risk of foetal zinc deficiency. This is because zinc may become trapped in the placenta leading to a decreased transfer to the foetus and an increased risk of impaired growth [45]. 

It is concerning to note that only 20.7% of women are meeting the recommended daily intake of magnesium. Magnesium is one of the essential nutrients that the body must acquire through exogenous sources, mainly through dietary intake. Its role in various biochemical and physiological processes is pivotal, and a deficiency can result in complex effects, ranging in severity. Bone formation is dependent on magnesium, which favours calcium assimilation and contributes to vitamin D activation [46]. Unfortunately, due to overprocessed food and carbohydrate refinement, a significant amount of magnesium is removed. Meeting the recommended intake is now more challenging than ever due to a plethora of agronomic and environmental factors [47]. Another aspect to take into account is the decrease in minerals of common vegetables consecutive to changes in cultivation practices [48]. Hypomagnesemia during pregnancy is associated with pre-eclampsia and pre-term birth [49]. Maternal magnesium deficiency can have long-lasting effects on the offspring’s health, including in paediatric stages [50]. Given the difficulty of establishing the presence of a magnesium deficit [51], coupled with its essential role in assuring a healthy foetus development, it is imperative to encourage a high intake of magnesium-rich foods during pregnancy. Animal studies also documented the adverse effects of maternal magnesium deficiency. In rats, hypomagnesemia during pregnancy resulted in increased body fat, insulin resistance and impaired glucose tolerance of the offspring [52]. Furthermore, the consequences of magnesium deficiency may extend beyond physical issues. In mice, maternal magnesium deficiency resulted in anxious behavioural patterns in their offspring, which persisted into adulthood [53].

Only 29.9% of women reached the recommended intake of Vitamin A in the form of Retinol Equivalents (REs), with the prudent pattern being the most likely to meet the requirements. Vitamin A intake has double-edged implications for pregnancy. Vitamin A is essential to ensure the normal implantation and consequent development of the embryo [54]. Vitamin A deficiency during pregnancy targets most of the organ systems of the foetus [55], causing complications for both the mother and the foetus [56]. However, vitamin A is mostly known for its teratogenic effects when ingested in excessive doses [57]. Given the deleterious effects caused by toxic levels of vitamin A, there seems to be a prevailing association with its potential harm, often obscuring the acknowledgement of its essential roles in assuring healthy foetus development. Targeted nutritional interventions could educate pregnant women on the important role of dietary vitamin A.

Lack of compliance with dietary guidelines was observed for vitamin C, with only 31.9% of participants meeting the recommended intake. Similarly, 47% of participants failed to meet the recommended level for vitamin E intake. Vitamin E is a powerful lipophilic antioxidant, essential for normal reproductive physiology. Moreover, several animal studies have observed placental discrimination for natural vitamin E, rather than synthetic [58]. Vitamin C, a water-soluble antioxidant, also plays an important role in the metabolism of iron and folate, and in the synthesis of collagen. Together, they act synergistically to help neutralise free radicals and protect against oxidative stress. A higher dietary intake of vitamin C was independently associated with reduced risks of gestational diabetes [59] and increased infant growth up to the age of 6 months [60]. High intakes of both vitamin C and E may also reduce the risk of severe pre-eclampsia and eclampsia [61]. It is important to consume adequate doses of both vitamin C and vitamin E. Vitamin C acts as a co-antioxidant and prevents vitamin E from facilitating lipid peroxidation in LDL particles [62]. Women following a prudent dietary pattern were also more likely to meet recommended polyunsaturated fatty acids (PUFAs) requirements. In the overall sample, 61.3% met the target intake. Given that PUFAs are essential for foetal growth and proper neurological and retinal development, proper maternal intake is crucial, as they can be acquired by the foetus only from maternal sources [63]. 

A major limitation of this study is that although a power sample analysis was conducted, it may not be sufficient to draw definitive conclusions about the entire population. Both social and educational backgrounds play an important role in ensuring compliance of the expectant mothers to dietary recommendations. One strength of our study consists of using both FFQ-derived dietary patterns and dietary quality indexes. Both these methods have proven reliable and informative in regards to pregnant women’s diets. Food frequency questionnaires (FFQs) are useful tools for measuring nutritional exposure and require less labour and economic resources than other types of nutritional investigations. Participant burden is also greatly reduced, as compared to methods such as weighted food journals, thus allowing for greater response rates. Nonetheless, several other limitations consist of reliance upon participants’ memory for food data recalling and requiring great interviewer experience and expertise to ensure accurate data collection. When referring to pregnant women in particular, research has shown that PCA of FFQs produces similar information as compared to food diaries, confirming its value [64]. Our sample was also limited to the North-Eastern region of the country. Future research should expand to include other regions of our country. 

There is a lack of data regarding the nutrition of pregnant women in Romania, as only a small number of studies have examined their dietary patterns [65,66]. As a result, this research serves as a valuable addition to the current literature and aids in enhancing our comprehension of this particularly important population group. Further research is necessary to ascertain the underlying factors driving these patterns and how population-based interventions can improve the quality of women’s nutrition.

## 5. Conclusions

The findings of our study demonstrate a significant relationship between dietary patterns and the quality of diet, assessed using the Diet Quality Index for Pregnancy (DQI-Pc), PURE Healthy Diet Score, and FIGO Diet Quality Score, with women with a prudent dietary pattern being more likely to adhere to a diet with a higher quality score. Moreover, we also identified a relationship between dietary patterns and specific nutrient intakes recommended by the European Food Safety Authority, such as B vitamins, folic acid and PUFAs. Further studies are needed to evaluate how dietary patterns and quality diet indices relate to maternal and offspring outcomes. Although we showcased the patterns associated with better nutrient intakes, additional research ought to link these patterns with the knowledge, attitudes and practices of pregnant women and also seek to increase awareness of the importance of diet during pregnancy.

## Figures and Tables

**Table 1:** Dietary patterns identified in the study sample through principal component analysis.

Food Groups	Dietary Pattern		
	Prudent	Modern	Vegetarian
Vegetables	**0.748**	0.225	−0.171
Fruit	**0.601**	0.077	0.017
Soups and sauces	**0.586**	0.264	−0.035
Milk and milk products	**0.520**	−0.190	**0.437**
Fish and fish products	0.160	−0.015	−0.004
Potatoes	−0.021	**0.605**	0.193
Fats and oils	−0.114	**0.587**	**0.544**
Non-alcoholic beverages	0.204	**0.563**	0.088
Meat and meat products	0.213	**0.549**	−0.075
Sugars, preserves and snacks	−0.019	**0.383**	−0.161
Cereals and cereal products	0.080	0.247	**0.719**
Eggs and egg dishes	0.236	0.096	−0.445
Nuts and seeds	0.375	−0.328	**0.385**

Variables bolded have an Eigenvalue > 0.3.

**Table 2:** Sociodemographic characteristics.

Parameter		N	%
Area of residence	urban	103	41
	rural	148	59
Education level	elementary school	19	7.6
	secondary school	85	33.9
	high-school	79	31.5
	university	68	27.1
Smoke status	no	167	66.5
	yes	58	23.1
	former	26	10.4
Diet advice	yes	37	14.7
	no	214	85.3
Folic acid supplements	yes	20	8
	no	231	92
Other supplements	yes	84	33.5
	no	167	66.5
BMI	normal weight	142	58.19
	overweight	55	22.54
	obese	47	19.26
Gestational weight gain	adequate	150	61
	inadequate	39	15.9
	excessive	57	23.2
Dietary patterns	Prudent	83	33.1
	Modern	90	68.9
	Vegetarian	78	31.1

**Table 3:** Anthropometric and gestational characteristics.

						Percentiles		
	Mean	Median	SD	Minimum	Maximum	25th	50th	75th
Age (years old)	26.55	26	5.44	18	40	22.5	26	30
Pre-gestational BMI (kg/m^2^)	25.06	24.35	5.24	15.42	45	21.1	24.35	27.59
Gestational weight gain (kg)	8.34	6.7	8.47	−8.1	41	2	6.7	12.23
No of pregnancies	1.73	1	0.94	1	5	1	1	2
Gestational age (weeks)	38.55	39	1.39	34	42	38	39	40
DQI-Pc score	37.29	36.43	8.82	18.28	65.15	30.73	36.43	41.88
DQI-Pc	53.3	52	12.6	26.1	93.1	44	52	59.7
PURE	2	2	0.97	0	5	1	2	3
FIGO-DQS	3.41	3	1.10	1	6	3	3	4

**Table 4:** Dietary quality index and PURE score—component descriptions.

DQI-Pc	Score Categories	*n*	%	PURE	Score Categories	*n*	%	FIGO	Score Categories	*n*	%
Cereals (% recommended servings) 6–11 servings of grains per day *	<50%	183	72.9	Vegetables (2–3 servings/day)	no	231	92	Meat and poultry (2–3/week)	no	15	6
	50–99%	67	26.7		yes	20	8		yes	236	94
	>100%	1	0.4	Fruits (2–3 servings/day)	no	196	78.1	Fruit and vegetables (2–3/day)	no	62	24.7
Fruits (% recommended servings) 3–5 servings of vegetables per day *	<50%	191	76.1		yes	55	21.9		yes	189	75.2
	50–99%	57	22.7	Legumes (3–4 servings/week)	no	48	19.1	Fish (1/week)	no	202	80.5
	>100%	3	1.2		yes	203	80.9		yes	49	19.5
Vegetables (% recommended servings) 2–4 servings of fruits per day *	<50%	172	68.5	Nuts or peanuts (7 servings/week)	no	248	98.8	Dairy (daily)	no	143	57
	50–99%	69	27.5		yes	3	1.2		yes	108	43
	>100%	10	4	Fish (2–3 servings/week)	no	218	86.9	Whole grain (≥1/day)	no	183	72.9
Folic acid (%RDA)	<50%	229	91.2		yes	33	13.1		yes	68	27.1
	50–99%	22	8.8	Dairy (14 servings/week)	no	63	25.1	Packaged foods (≤5/week)	no	161	64.1
Iron (%RDA)	<50%	200	79.7		yes	188	74.9		yes	90	35.9
	50–99%	17	6.8	PURE score	0	16	6.4	FIGO-DQS	1	13	5.2
	>100%	34	13.5		1	55	21.9		2	32	12.7
Calcium (%AI)	<50%	66	26.3		2	108	43		3	87	34.7
	50–99%	159	63.3		3	58	23.1		4	78	31.1
	>100%	26	10.4		4	13	5.2		5	39	15.5
Fats (%AI)	>100%	251	100		5	1	0.4		6	2	0.8

* Based on Food Guide Pyramid recommendations for diets containing <1600, 1601–1900, 1900–2500, 2500–2800 and ≥2800 kcal.

**Table 5:** Dietary intake and adequate nutrient intake.

						Percentiles			Adequate Intake	
	Mean	Median	SD	Min	Max	25th	50th	75th	Count	%
Energy (kcal)	1992.53	1927.44	537.29	913.58	3500	1610.00	1927.44	2274.12		
Protein	80.41	77.87	21.68	35.22	155.61	64.94	77.87	93.56	248	98.8
Carbohydrate	264.66	258.11	74.77	109.57	575.17	209.78	258.11	298.85	163	64.9
Fat total	75.27	70.86	25.85	30.48	221.17	58.63	70.86	88.73	249	99.2
MUFAs total	26.14	24.36	10.16	9.03	98.85	19.47	24.36	30.82	42	16.7
PUFAs total	14.54	13.46	6.14	3.96	65.26	10.66	13.46	16.64	154	61.3
SFAs total	27.75	26.15	10.17	9.71	61.89	20.59	26.15	33.40	38	15.1
Cholesterol	362.96	346.04	139.94	80.11	987.85	267.90	346.04	433.40	85	33.9
Starch	156.82	154.18	46.55	54.57	404.15	127.56	154.18	184.56	226	90.0
Calcium	812.39	779.39	318.21	222.24	1894.02	584.71	779.39	972.10	185	73.7
Copper	1.31	1.19	0.60	0.47	4.43	0.98	1.19	1.43	243	96.8
Iron	13.94	10.09	10.76	4.93	45.47	8.18	10.09	12.74	35	13.9
Selenium	77.68	76.93	21.99	28.03	187.31	63.16	76.93	89.69	198	78.9
Zinc	8.34	7.92	2.45	3.99	18.30	6.52	7.92	9.69	32	12.7
Iodine	234.13	253.03	92.28	48.79	466.45	144.93	253.03	298.09	174	69.3
Magnesium	251.13	239.62	70.40	107.68	578.37	201.76	239.62	286.93	52	20.7
Manganese	2.30	2.12	0.81	0.73	7.12	1.84	2.12	2.61	239	95.2
Sodium	2960.45	2923.41	984.57	1069.11	6199.50	2237.04	2923.41	3638.15	43	17.1
Niacin	19.92	19.34	6.23	7.86	42.57	15.98	19.34	23.22	212	84.5
Total folate	210.46	203.18	62.41	93.13	473.65	162.05	203.18	248.22	16	6.4
Vitamin A	755.94	361.65	975.60	45.49	5371.35	247.72	361.65	990.81	146	58.2
Vitamin A Retinol equivalents	1366.68	1104.72	1113.35	132.09	7195.70	605.97	1104.72	1639.47	75	29.9
Vitamin B2	1.53	1.42	0.59	0.49	3.68	1.08	1.42	1.85	130	51.8
Vitamin B1	1.32	1.28	0.40	0.53	2.72	1.02	1.28	1.57	95	37.8
Vitamin B12	4.94	4.01	3.78	0.44	24.78	2.71	4.01	6.09	205	81.7
Vitamin B6	1.89	1.80	0.56	0.77	4.41	1.50	1.80	2.25	106	42.2
Vitamin C	76.66	72.47	39.71	7.65	346.07	47.60	72.47	93.70	80	31.9
Vitamin D (UI)	616.03	855.98	383.37	10.11	1080.05	104.16	855.98	891.19	167	66.5
Vitamin E	12.55	11.85	4.73	3.05	31.08	9.18	11.85	14.62	118	47.0

**Table 6:** Relationship between dietary patterns and the quality of diet.

				95% Confidence Interval				95% Confidence Interval	
Predictor	Dietary Pattern	*p*	OR	Lower	Upper	*p*	aOR *	Lower	Upper
Calcium	1–3	0.1	1.79	0.894	3.59	0.071	1.93	0.946	3.95
	2–3	0.09	1.81	0.912	3.59	0.055	1.99	0.986	4.02
	1–2	0.978	0.99	0.484	2.03	0.939	0.972	0.465	2.03
Magnesium	**1–3**	**0.027**	**1.085**	**0.735**	**3.909**	**0.015**	**2.327**	**1.18**	**4.59**
	**2–3**	**0.002**	**1.47**	**0.256**	**0.921**	**<0.001**	**3.136**	**1.594**	**6.17**
	1–2	0.334	0.741	0.404	1.361	0.36	0.742	0.392	1.406
Sodium	**1–3**	**0.003**	**4.02**	**1.597**	**10.11**	**0.003**	**4.03**	**1.593**	**10.2**
	2–3	0.124	1.8	0.851	3.81	0.116	1.839	0.86	3.94
	1–2	0.099	2.232	0.86	5.79	0.11	2.191	0.838	5.73
Niacin	**1–3**	**0.017**	**2.85**	**1.2**	**6.72**	**0.013**	**3.178**	**1.279**	**7.89**
	**2–3**	**0.019**	**2.7**	**1.17**	**6.21**	**0.02**	**2.777**	**1.179**	**6.55**
	1–2	0.915	1.053	0.405	2.739	0.792	1.144	0.42	3.114
Vitamin A	**1–3**	**<0.001**	**3.607**	**1.862**	**6.99**	**<0.001**	**4.16**	**2.073**	**8.35**
	**2–3**	**0.043**	**1.9**	**1.021**	**3.54**	**0.027**	**2.085**	**1.087**	**4**
	1–2	0.051	1.898	0.998	3.609	**0.043**	**1.995**	**1.021**	**3.9**
Vitamin A retinol	**1–3**	**0.01**	**2.5**	**1.248**	**5.009**	**0.008**	**2.645**	**1.296**	**5.4**
	2–3	0.971	0.986	0.47	2.068	0.988	0.994	0.466	2.12
	**1–2**	**0.007**	**2.535**	**1.295**	**4.964**	**0.006**	**2.66**	**1.331**	**5.32**
Vitamin B2	1–3	0.124	1.328	0.711	2.48	0.258	1.464	0.757	2.831
	2–3	0.243	1.631	0.879	3.03	0.088	1.766	0.918	3.396
	1–2	0.506	0.814	0.444	1.49	0.565	0.829	0.437	1.572
Selenium	1–3	0.679	1.82	0.849	3.91	0.075	2.048	0.931	4.5
	2–3	0.243	1.54	0.744	3.2	0.164	1.699	0.805	3.59
	1–2	0.679	1.213	0.54	2.58	0.65	1.205	0.538	2.7
Vitamin B1	**1–3**	**0.002**	**2.955**	**1.496**	**5.836**	**0.016**	**2.489**	**1.187**	**5.22**
	**2–3**	**0.009**	**2.436**	**1.243**	**4.773**	**<0.001**	**4.391**	**2.12**	**9.09**
	1–2	0.529	1.774	0.665	2.211	0.082	0.567	0.299	1.075
Vitamin B12	**1–3**	**0.012**	**2.87**	**1.26**	**6.54**	0.659	1.198	0.537	2.67
	2–3	0.049	2.13	1	4.53	0.437	1.377	0.615	3.08
	1–2	0.506	1.345	0.562	3.219	7.37	0.87	0.387	1.96
Vitamin B6	**1–3**	**0.004**	**2.65**	**1.37**	**5.126**	**0.022**	**2.253**	**1.125**	**4.511**
	**2–3**	**0.004**	**2.596**	**1.357**	**4.968**	**0.006**	**2.633**	**1.329**	**5.218**
	1–2	0.472	0.801	0.437	1.467	0.629	0.856	0.454	1.612
Vitamin C	**1–3**	**<0.001**	**6.795**	**3.0004**	**15.39**	**0.001**	**3.564**	**1.642**	**7.73**
	**2–3**	**<0.001**	**4.23**	**1.8671**	**9.583**	**0.002**	**3.356**	**1.558**	**7.23**
	1–2	0.987	0.995	0.534	1.853	0.853	1.062	0.562	2.008
Vitamin E	1–3	0.516	0.812	0.433	1.52	**0.042**	**1.964**	**1.024**	**3.77**
	2–3	0.067	1.771	0.96	3.27	**0.011**	**2.31**	**1.21**	**4.41**
	1–2	0.601	0.851	0.465	1.557	0.609	0.85	0.457	1.583
Zinc	1–3	0.099	2.237	0.8594	5.825	0.113	2.519	0.802	7.907
	2–3	0.648	1.2679	0.4584	3.507	0.113	2.498	0.805	7.753
	1–2	0.943	0.968	0.4016	2.335	0.986	1.01	0.402	2.531
Starch	**1–3**	**0.006**	**0.168**	**0.0467**	**0.6**	**0.037**	**3.56**	**1.081**	**11.72**
	2–3	0.424	0.56	0.1353	2.318	0.32	1.62	0.626	4.19
	1–2	0.192	2.25	0.665	7.61	0.213	2.198	0.637	7.58
SFAs	1–3	0.088	0.448	0.178	1.125	0.859	0.92	0.368	2.3
	2–3	0.665	0.84	0.381	1.851	0.723	1.17	0.491	2.78
	1–2	0.644	0.823	0.36	1.882	0.586	0.787	0.332	1.86
MUFAs	1–3	0.864	0.548	0.333	1.793	0.814	0.902	0.382	2.13
	2–3	0.854	0.977	0.448	2.181	0.509	0.749	0.318	1.77
	1–2	0.864	1.074	0.477	2.416	0.668	1.204	0.515	2.81
PUFAs	**1–3**	**0.041**	**1.935**	**1.026**	**3.65**	0.684	8.71	0.448	1.69
	2–3	0.629	0.853	0.447	1.627	0.79	1.09	0.564	2.12
	1–2	0.644	0.864	0.465	1.61	0.492	0.796	0.416	1.52
Iodine	1–3	0.07	1.912	0.949	3.85	0.807	1.10	0.52	2.314
	2–3	0.899	0.96	0.508	1.81	0.849	1.073	0.516	2.231
	1–2	0.046	1.99	1.012	3.92	0.952	1.022	0.498	2.1
Folic acid (as folate)	**1–3**	**0.037**	**5.205**	**1.1028**	**24.569**	**0.018**	**2.794**	**1.197**	**6.52**
	2–3	0.518	1.767	0.3148	9.922	**0.034**	**2.477**	**1.072**	**5.727**
	1–2	0.078	0.34	0.102	1.13	0.741	1.128	0.553	2.301
Iron	1–3	0.698	0.849	0.371	1.942	0.719	1.165	0.506	2.68
	2–3	0.411	0.703	0.304	1.627	0.272	0.594	0.235	1.5
	1–2	0.664	0.828	0.355	1.94	0.145	1.962	0.793	4.85
Vitamin D	1–3	0.265	1.461	0.75	2.85	0.91	0.958	0.456	2.013
	2–3	0.918	0.967	0.515	1.82	0.658	0.849	0.411	1.753
	1–2	0.21	1.51	0.793	2.88	0.738	1.129	0.555	2.294
Cholesterol	**1–3**	**0.004**	**0.386**	**0.202**	**0.74**	**0.028**	**0.467**	**0.237**	**0.923**
	**2–3**	**<0.001**	**0.289**	**0.149**	**0.559**	**0.041**	**0.499**	**0.256**	**0.971**
	1–2	0.803	0.92	0.476	1.775	0.852	9.37	0.474	1.852
DQI-P (Q75)	**1–3**	**<0.001**	**4.812**	**2.355**	**9.833**	**<0.001**	**4.932**	**2.307**	**10.54**
	2–3	0.614	1.253	0.5222	3.005	0.132	1.854	0.831	4.14
	**1–2**	**<0.001**	**6.027**	**2.7314**	**13.3**	**<0.001**	**6.653**	**2.868**	**15.43**
PURE Q75	**1–3**	**<0.001**	**9.532**	**4.204**	**21.60**	**0.001**	**9.68**	**4.152**	**22.605**
	2–3	0.193	1.785	0.74	4.27	0.139	1.956	0.804	4.759
	**1–2**	**<0.001**	**5.339**	**2.68**	**10.56**	**<0.001**	**4.951**	**2.435**	**10.065**
FIGO Q75	**1–3**	**<0.001**	**4.648**	**2.392**	**9.031**	**<0.001**	**5.001**	**2.514**	**9.95**
	2–3	0.226	1.482	0.785	2.791	0.217	1.498	0.788	2.85
	**1–2**	**<0.001**	**3.140**	**1.680**	**5.87**	**<0.001**	**3.338**	**1.738**	**6.41**

OR—odds ratio, aOR—adjusted odds ratio, * adjusted for age, formal education, income, area of residence, parity, and the presence of nutritional advice during pregnancy; 1—prudent pattern; 2—modern pattern; 3—vegetarian pattern; values bolded have *p* < 0.05

## Data Availability

The original contributions presented in the study are included in the article, further inquiries can be directed to the corresponding author.
